# Changing Epidemiology of Hepatocellular Adenoma in the United States: Review of the Literature

**DOI:** 10.1155/2013/604860

**Published:** 2013-02-24

**Authors:** Charissa Y. Chang, Juan C. Hernandez-Prera, Sasan Roayaie, Myron Schwartz, Swan N. Thung

**Affiliations:** ^1^Division of Liver Diseases, The Mount Sinai Medical Center, One Gustave Levy Place, P.O. Box 1104, New York, NY 10029-6574, USA; ^2^Recanati/Miller Transplant Institute, The Mount Sinai Medical Center, New York, NY, USA; ^3^Department of Pathology, The Mount Sinai Medical Center, New York, NY, USA

## Abstract

Hepatocellular adenoma (HCA) is a benign neoplasm arising from hepatocytes. There is evidence that the inflammatory subtype may be associated with obesity and alcohol use and that men with metabolic syndrome may be at risk for malignant transformation of HCA. We sought to explore the combined experience of US centers as reported in the literature to document the epidemiologic shift in risk factors for HCA formation in the United States, namely, a shift from oral contraceptive pills (OCPs) to an emerging role of obesity as a contributing factor. *Methods*. Publications reporting HCA in the United States were identified through a PubMed search and a review of the literature. We excluded publications prior to 1970, single case reports, and publications for which there was no data available regarding patient characteristics including OCP use and the number of adenomas. *Conclusion*. Whereas earlier reports of HCA in the United States described cases exclusively in women exposed to OCPs, there is a trend towards an increase in HCAs reported in men, HCAs in the absence of OCP use, and increased reporting of multiple HCAs. This may be a result of newer OCP formulations and increasing prevalence of obesity.

## 1. Introduction

Hepatocellular adenomas (HCAs) are benign hepatic neoplasms that became widely recognized in the 1960s and 1970s following the introduction of oral contraceptive pills (OCP's). Recent advances have identified distinct subtypes based on genotypic classification [1]. These types are (1) hepatocyte nuclear factor-1*α* (HNF-1*α*)-mutated HCAs (H-HCA), (2) *β*-catenin-mutated HCAs (b-HCA), (3) inflammatory HCAs (I-HCA) (which harbor mutations involving the interleukin-6 signal transducer), and (4) unclassified. I-HCAs and H-HCAs account for the majority (80%), while b-HCAs comprise about 10%–15% [2]. Ten percent of I-HCAs also demonstrate *β*-catenin mutation; however, H-HCAs and b-HCAs are mutually exclusive [3]. 

HCAs appear as unencapsulated tumors that may be solitary or multiple. Adenomatosis, a term used when greater than 10 adenomas are encountered, can be associated with maturity onset diabetes of the young type 3 (MODY3). Histologically, HCAs are characterized by plates of hepatocytes that lack portal tract elements and are separated by sinusoids. Immunohistochemical staining techniques proposed by the Bordeaux group [1] and validated by others [4–6] aid in the classification of HCAs into the different subtypes which are reviewed briefly in the following.

H-HCAs result from inactivating mutations in the hepatocyte nuclear factor 1 A (HNF1A) gene. Histologic features include marked hepatocellular steatosis, lack of cytologic atypia, and absence of inflammatory infiltrates. This subtype occurs almost exclusively in women and can also be associated with maturity onset diabetes of the young type 3 (MODY3), a condition caused by germline mutations in HNF1A. IHC staining distinguishes this subtype through absent expression of liver fatty acid binding protein (LFABP) in tumoral hepatocytes and normal expression in nontumoral liver.

b-HCAs are characterized histologically by nuclear atypia and pseudoacinar formation. This subtype has been associated with men, androgen treatment, and glycogen storage disease. IHC demonstrates aberrant nuclear beta-catenin staining and strong positive staining for glutamine synthetase. This subtype has been associated with an increased risk of malignant transformation [2]. Twenty to thirty percent of HCAs undergoing malignant transformation show *β*-catenin mutations [1, 7].

Inflammatory HCAs comprise 40%–50% of adenomas and are the type most commonly associated with OCP use, although obesity and alcohol consumption have also been identified as risk factors [1]. Histologic features include sinusoidal dilatation, inflammatory infiltrates, peliosis, and pseudoportal tracts with thickened arteries and which lack veins and ducts [3]. Prominent ductular reaction may be present. IHC reveals expression of serum amyloid-associated protein A2 (SAA-2) and C-reactive protein. I-HCAs have not been found to be associated with malignant transformation [3].

With the advent of modern imaging, most adenomas that come to attention are discovered incidentally. Patients may also present with abdominal pain or a palpable mass; hemorrhage is a presenting feature in 20%–40%, and malignant transformation is estimated to occur in 4%–10% [17]. For patients with an asymptomatic solitary adenoma, size greater than 5 cm is commonly considered a basis for elective surgical resection to preempt the risk of bleeding or cancer.

### 1.1. OCPs as a Risk Factor for HCA

OCPs were first introduced in 1960 and initially contained concentrations of estrogen and progestin that were 2–5 times and 5–10 times higher than current formulations [24]. Before long, an association between OCP use and the development of HCA came to light [21, 23]. In 1979, a case-control study from the Armed Forces Institute of Pathology database estimated an annual incidence of 3-4 HCA per 100,000 long-term (>24 months) users of OCPs as opposed to 0.13 per 100,000 in nonusers [20]. Other complications of early generation OCPs (most notably cardiovascular/thromboembolic) were even more problematic, and with refined understanding of the physiology behind OCPs, subsequent formulations with lower hormonal concentrations were rapidly introduced [24] resulting in a markedly decreased incidence of OCP-associated HCAs [25]. Estrogen levels in women on modern-day OCPs are not higher than normal physiologic levels, and the ongoing association of HCAs with OCPs is primarily the result of the ubiquity of OCP use; indeed, it is hard to find women of child-bearing age who have not used OCPs.

### 1.2. Role of Obesity and Metabolic Syndrome

New risk factors for HCA have emerged in recent years, in particular obesity [9, 26] and metabolic syndrome [5]. Farges et al. demonstrated an increase in incident malignant HCAs in men over a 15-year period whereas the number of HCAs with malignancy in women did not change over time. Six of twelve men with HCA in their series had metabolic syndrome [5]. Bioulac-Sage reported similar findings in their experience-with cases of HCA that presented to a single center over a 20 year period, namely, an increase in overweight/obese men presenting with HCA [26]. 

I-HCA is associated with obesity and with steatosis in the nontumoral liver. This was first suggested by Paradis et al. who found that most cases of I-HCA (which in the past was called telangiectatic focal nodular hyperplasia) occurred in overweight or obese patients and that steatosis outside of tumors was found in 69% of cases, with moderate/severe steatosis in 30% [27]. The association between steatosis and HCA was confirmed by several other groups [9, 10] including case reports of HCA [28] and adenomatosis [29–31] occurring in the background of nonalcoholic steatohepatitis. 

### 1.3. Adenomatosis and Hepatic Steatosis

Vetalainen reported a review of 94 cases of adenomatosis in the literature and found that 18% had steatosis [31]. Another study [32] found that hepatic steatosis as measured by CT scan was present in 82% of patients with multiple HCAs as compared with 58% of patients with single HCA and 29% in a control group of patients with hemangioma. These series highlight the association between HCAs (in particular multiple HCAs) and steatosis and suggest potential factors that drive both steatosis and formation of HCAs.

We reviewed the combined experience of US centers as reported in the literature to identify whether there is an epidemiologic shift in risk factors for HCA from OCP use to obesity and metabolic syndrome and whether there is a resultant increasing incidence of HCAs in men. 

## 2. Case Series Describing HCA in the United States

Several case series, most of which are single-center experiences, report clinical characteristics of HCA in the United States. Most of the series are single-center experiences from surgical or pathologic databases of resection specimens and are therefore subject to selection bias. Publications reporting HCA in the United States from 1970 onwards were identified through a PubMed search and review of the literature. Published series for which information regarding clinical presentation was available were selected and divided into “early experience” (prior to 1985) and “later experience” (beyond 1985), as summarized in Tables 1 and 2. Whereas earlier case series tended to report single HCAs, nearly all found in women taking OCPs, later series describe more cases presenting with multiple HCAs, more men, and fewer cases associated with OCP use. We explore this in more detail later and highlight recent evidence supporting an emerging role of obesity and metabolic syndrome as a possible factor to explain cases of HCA in the absence of hormonal therapy. 

The two largest surgical series of HCA experiences from the United States are a multicenter series [11] and the Pittsburgh experience [12]. Deneve et al. [11] described 124 patients with HCA from 5 different hepatobiliary centers over a nine-year period with an aim to identify risk factors associated with rupture and/or malignant transformation. All of the patients in the series received treatment; the majority (96%) underwent resection while the rest (4%) were treated with embolization. Of those who underwent resection, 25% had evidence of rupture. Hormone use was more common in ruptured versus nonruptured HCA (58% versus 25%, *P* = 0.001). Ruptured HCAs were also larger (10.5 ± 4.5 cm versus 7.2 ± 4.8 cm). Logistic regression analysis identified tumor size >7 cm (*P* = 0.001) and hormone use (*P* = 0.007) as independent risk factors for rupture. No ruptured HCA was <5 cm. Five patients (4%) had HCC; all occurred in HCAs that were >8 cm, and three of five occurred in patients who were taking OCPs. Mean BMI was 27 (±5), and there was no correlation between BMI and risk for rupture. History of hormone use was reported in 55% of patients, which the authors noted was lower than rates of 72%–77% reported in earlier series. Details about patients who developed HCA in the absence of OCP use were not available.

Cho et al. published a single center surgical series from Pittsburgh describing their experience with 41 patients who underwent resection for HCA over a 19-year period [12]. The primary aim was to identify factors which correlate with bleeding and the development of cancer. Most patients (93%) were women, and the median age was 36 years. OCP use was reported in 22 women (54%). Five patients had evidence of steatosis; information on BMI, metabolic syndrome, or other potential risk factors in the individuals diagnosed with HCA in the absence of OCP use was not provided. Rates of hemorrhage and malignancy were 29% and 4.8%, respectively. Both of these series were published before molecular classification of HCA subtypes was available; therefore there is no information provided regarding molecular classification.

### 2.1. Recent Series Highlighting the Role of Obesity

Recently, Bunchorntavakul et al. described a single-center experience of 60 consecutive patients with HCA over a 5-year period [9]. The primary aim of this study was to investigate whether there is a correlation between obesity and the clinical course of HCAs. Unlike other series which were based entirely on surgical specimens, this series included both patients who underwent resection and patients who were observed in the absence of intervention over a median followup of 2.4 years. Diagnosis in nonsurgical cases was based on imaging and clinical features; pathology was available in 32 patients. Of these, five (16%) had I-HCA. Steatosis and steatohepatitis were found in the nontumoral liver in 28% and 16% of cases, respectively. Eleven (18%) patients were overweight and 33 (55%) were obese. In comparison, the authors note that this is higher than the prevalence of obesity of 27%–34% in an age- and sex-matched population in the same region. Obesity was associated with multiple adenomas (85 versus 48%, *P* = 0.007), bilobar distribution (67% versus 33%, *P* = 0.01), and tumor size progression (33% versus 0%, *P* = 0.05). Unlike other groups, the authors did not note an association between obesity and I-HCA. 

The 26 patients who were observed over a median 2.4 years (1–7.3 years) provide a view, albeit limited, of the natural history of HCA. Resection was advised for HCAs >5 cm, bleeding, or inability to exclude malignancy; asymptomatic HCAs <5 cm were imaged every 6–12 months. Patients taking OCPs were advised to stop. HCAs remained stable in 77% and regressed in 4%; progression was observed in 19%, with progression more likely in obese patients (33.3% versus 0, *P* = 0.05). In summary, this study demonstrates an association between obesity and multiple adenomas as well as progression of adenomas. 

**Figure 1:**
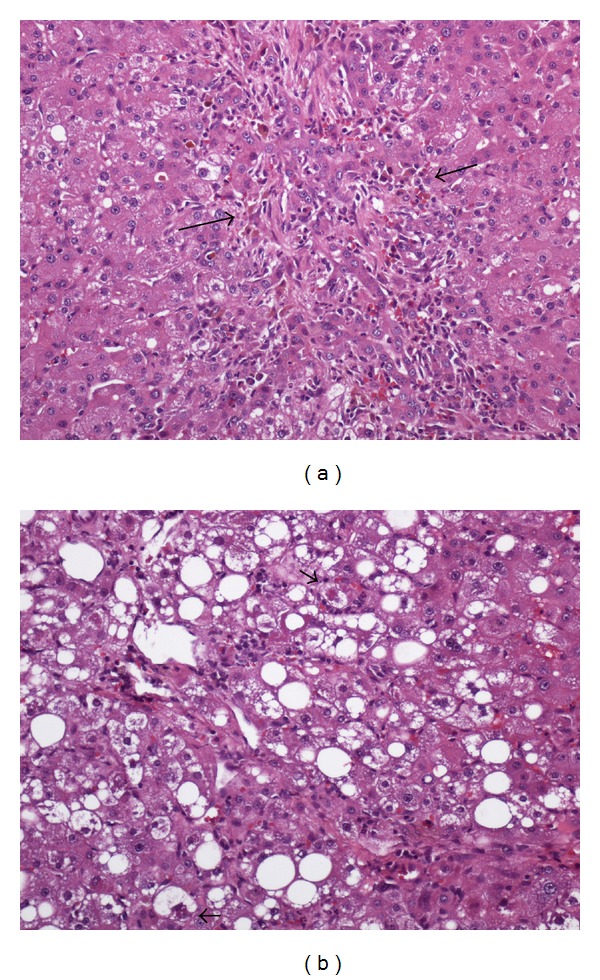
A 40-year-old man with NASH with severe activity (grade 3 of 3) and transition to cirrhosis (stage 3 of 4). (a) Hepatocellular adenoma containing portal tract-like structures with mild inflammation and marked ductular reaction (arrows). Steatosis is absent (H&E stain, ×100). (b) Nontumoral liver with severe ballooning degeneration of hepatocytes. Some of the ballooned hepatocytes contain Mallory-Denk hyalines (H&E stain, ×100).

Two case reports describe hepatic adenomatosis in the setting of steatohepatitis [29, 30] in the USA. Brunt described a 54-year-old woman with elevated BMI and prior OCP exposure who presented with abdominal pain and adenomatosis. The second case, described by our center, highlights a 53-year-old woman with BMI 24 and OCP use who presented with adenomatosis and background of moderate steatohepatitis. IHC showed positive SAA staining consistent with I-HCA.  Figure 1 illustrates a case from our center of a 40 year old man with HCA arising in the background of nonalcoholic steatohepatitis (NASH). These along with the series from Bunchorntavakul hint at a rising incidence of HCA in the setting of obesity and/or steatohepatitis.

## 3. Comparison of the US Experience in relation to the World

There are single-center series from France [5, 26] demonstrating a rising incidence of HCA in men. There are no single-center US series showing this, but comparison of published series from 1970 to 1985 shown in Table 2 with the series from 1985 to 2011 shown in Table 1 reveals a trend similar to what has been described in Europe. HCAs in men were rarely reported in the early series published from 1970 to 1985 (Table 2) whereas the later series published from 1985 to 2011 describe more male patients and more malignancies. This discrepancy cannot be explained entirely by the introduction of molecular and immunohistochemical studies which were introduced after 2006. One hypothesis is that increasing prevalence of obesity may play a role in this epidemiologic shift towards incident cases of HCA in men. Interestingly, studies from both China [33] and Japan [6] report a preponderance of HCA in men, which may reflect different practices with OCP use. The studies included patients with Hepatitis B; therefore some cases which were classified as HCA may in fact have been well-differentiated HCC.

The reader is directed to the study by Balabaud et al. in this volume which describes molecular classification of HCAs from different centers around the world. The Mount Sinai experience of 61 resected HCAs cases over a 12-year period (1990–2012) shows a preponderance of I-HCA (44%) followed by H-HCA (33%) and unclassified HCA (16%) and only one case (2%) of b-HCA (unpublished data). This is a lower proportion of b-HCA cases than what has been described in Europe (10%–15%). One case that had features of both I-HCA and b-HCA had a focus of hepatocellular carcinoma.

## 4. Conclusions

There appears to be a rising incidence of HCA diagnosis as reflected in the trend towards publication of larger case series over recent years, though this is due in large part to increasing discovery of incidental HCAs with widespread use of imaging. Alternatively, it could be that with time, centers have accrued a larger population to report.

The impact of modern OCPs on the development of HCA is probably marginal; on the other hand, in concordance with reports from European groups, there is increasing evidence to suggest obesity and metabolic syndrome as emerging risk factors for HCA. Multiple HCAs/adenomatosis in association with these risk factors appear to be increasing as suggested by case reports in the literature. Furthermore, European groups [5, 26] have highlighted recent cases of malignant transformation of HCA in men with metabolic syndrome. 

The prevalence of obesity in the USA has risen dramatically from around 14% in the 1970s to over 35% now [34, 35]. Nonalcoholic fatty liver disease (NAFLD) is now recognized as the hepatic manifestation of metabolic syndrome and is emerging as a common cause of chronic liver disease in parallel with rising obesity trends. It is unclear whether the shift in epidemiology of HCAs and recent reports of HCA in the setting of steatohepatitis are related directly to obesity and NAFLD or whether this merely reflects coincident epidemiologic changes in the population at large. One potential mechanism linking obesity with HCA formation includes increased levels of adipokines such as IL-6. This could lead to formation of inflammatory HCAs which have been found to demonstrate increased activation of the IL-6 signalling pathway [36]. Further studies demonstrating an association between I-HCA and obesity would support this hypothesis. Other mechanisms which could link obesity with HCA formation include the hyperestrogen state that is associated with obesity.

Additional single-center or multicenter longitudinal studies that include both surgical and nonsurgical cases are warranted to examine the evolution of risk factors for HCA in the United States. Future studies should include BMI and metabolic syndrome in descriptions of cases, focusing on both men and women, and should include data on IHC staining to facilitate molecular classification. 

## Figures and Tables

**Table 1:** Published series of hepatocellular adenoma in the United States (1985–2011).

Author Year of publication Study duration	n	Sample population	Hormonal therapy (%)	Women (%)	Mean age	Single	Multiple	Presenting symptoms	Hemorrhage	Malignancy	Treatment
Deodhar et al. [8] 2011 (2006-2007)	8	Single-center series, bland embolization	4/8 (50%)	7/8 (87.5%)	39	1 (12.5%)	7 (87.5%)	6 (75%) asymptomatic 1 (12.5%) rupture 1 (12.5%) abdominal pain	1/8 (12.5%)	0	Bland embolization (all)
Bunchorntavakul et al. [9] 2011 (2005–2010)	60	Single-center series	45 (75%)	58 (97%)	36 (median)	28%	72%	36 (60%) incidental 15 (25%) abdominal pain 7 (11.7%) bleeding 2 (3.3%) abnormal LFTs	7 (11.6%)	0	17 resection 9 TAE 8 sequential TAE + resection 26 observation
Mounajjed and Wu [10] 2011 (1994–2010)	35	Single-center series from pathology database	23/29 (79%)	30 (86%)	39	15 (43%)	20 (57%)	NR	10/49 (20%)	0	Resection (all)
Deneve et al. [11] 2009 (1997–2006)	124	Multicenter surgical series	55%	116 (94%)	39	77 (62%)	47 (38%)	NR	31 (25%)	5 (4%)	119 resection 5 embolization 8 RFA (during resection)
Cho et al. [12] 2008 (1988–2007)	41	Single-center surgical series	22 (54%)	38 (93%)	36 (median)	NR	NR	29 (70%) abdominal pain 7 (17%) incidental (on imaging) 3 (7%) abnormal LFTs 2 (5%) incidental (laparoscopy)	12 (29%)	2 (4.8%)	Resection (all)
Charny et al. [13] 2001 (1992–1999)	12	Surgical series	NR	10 (83%)	34	NR	NR	8 (67%) “symptoms” 3 (25%) abnormal LFTs	NR	1/8	8 resection
Reddy [14] 2001 (1983–1997)	25	Single-center series, database of cases from radiologic or surgical diagnosis	21/22 (95%)^1^	25 (100%)	33	16 (64%)	9 (36%)	3 (12%) rupture	3 (12%)	1 (4%)	19 resection 1 unresectable 2 no intervention
Ault et al. [15] 1996 (1985–1995)	11	Single-center surgical series	9/11 (82%)	10 (91%)	37.6	8 (73%)	3 (27%)	10 (91%) abdominal pain	4 (36%)	3 (27.2%)	4 resection 4 observation 4 embolization
Nagorney [16] 1995 (1989–1993)	24	Single-center series	9/22 (41%)	22 (92%)	36	20 (83%)	4 (17%)	19 (79%) symptomatic	4	2^2^	19 resection 4 observation 1 liver transplantation

TAE: transarterial embolization, NR: not reported.

^1^No data on OCP use in 3.

^2^One HCC, one intrahepatic cholangiocarcinoma.

**Table 2:** Published series of hepatocellular adenoma in the United States (1973–1985).

Author Year of publication Study duration	*n*	Sample population	Hormonal therapy (%)	Women (%)	Mean age	Single	Multiple	Presenting symptoms	Hemorrhage	Malignancy	Treatment
Mays and Christopherson [18] 1984 (1973–1984)	71	Single-center surgical registry	83%	100%	30	NR	NR	(36.6%) mass (31%) hemoperitoneum (24%) pain (5.6%) incidental (2.8%) unknown	NR	NR	
Weil et al. [19] 1979 (1970–1978)	8	Single-center surgical series	4 (50%)	7 (88%)	24	8 (100%)	0	6 fever 6 abdominal pain 3 palpable abdominal mass	4 (50%)	0	Resection
Bourne Rooks et al. [20] 1979 (1957–1976)	79	Database of pathology referrals	73 (92%)	79 (100%)	30.4	NR	NR	34% intraperitoneal bleeding 15% intratumoral hemorrhage 47% abdominal mass 19% abdominal pain	49%	0	Resection
Edmondson et al. [21] 1976 (1955–1976)	42	Single-center surgical series	31/36^1^ (86%)	42 (100%)	NR	NR	NR	12 (28%) pain 18 (43%) palpable mass 2 (4.7%) incidental	10 (24%)	0	41 resection
Ameriks et al. [22] 1975 (1969–1973)	8	Single-center surgical series	8 (100%)	8 (100%)	33.6	7	1	3 hemoperitoneum 3 abdominal pain, intrahepatic hemorrhage 3 palpable mass	6 (75%)	0	Resection
Baum et al. [23] 1973	7	Case series	7 (100%)	7 (100%)	28.7	7	0	5 abdominal pain 2 right upper quadrant mass	5	0	Resection

NR: not reported.

^1^Data on OCP use not available in 6 cases.
